# Solitary Screen Time Exacerbates Later Socioemotional Problems in Young Children with Oral Language Difficulties

**DOI:** 10.1007/s10802-025-01409-8

**Published:** 2026-03-12

**Authors:** Molly Selover, Mary Page Leggett-James, Anders Højen, Dorthe Bleses, Brett Laursen

**Affiliations:** 1 Department of Psychology, Florida Atlantic University, 777 Glades Road, Boca Raton, FL 33134 USA; 2 School of Communication and Culture, TrygFondens Centre for Child Research, Aarhus University, Aarhus, Denmark

**Keywords:** Solitary screen time, Oral language difficulties, Socioemotional outcomes, Home environment

## Abstract

Strong evidence ties early language difficulties to later adjustment challenges. Little is known, however, about factors that exacerbate these associations. The present study tests the hypothesis that unsupervised, solitary screen time amplifies longitudinal associations from low language skills to heightened socioemotional difficulties. The participants were 546 (264 girls, 282 boys) 4–5-year-olds attending 24 population-based childcare centers in 13 municipalities across Denmark. Teachers twice completed assessments of child adjustment difficulties (i.e., conduct problems and emotional problems), approximately six months apart. At the outset, teachers assayed child language abilities (i.e., communication skills and productive vocabulary) and parents reported solitary screen time (i.e., the amount of time children spent alone viewing handheld devices or television). Results indicated that solitary screen time and low communication skills predicted increases in subsequent emotional problems. Moderated associations emerged for conduct problems, such that solitary screen time exacerbated longitudinal associations from oral language problems to later adjustment difficulties. Specifically, among those with above (but not below) average levels of solitary screen time, low initial productive vocabulary and low initial communication skills predicted increases in conduct problems across the course of six months within a single school year.

**Supplementary Information:**

The online version contains supplementary material available at 10.1007/s10802-025-01409-8.

## Introduction

Early language difficulties forecast later socioemotional adjustment challenges. Meta-analytic findings indicate that language impairments during early childhood give rise to heightened internalizing and externalizing symptom severity two to twelve years later (Yew & O’Kearney, 2012). Oral language skills (e.g., speaking and understanding words, sentences or phrases) have been a particular area of focus, because communicative competence is increasingly expected of preschool children as their social world expands to include more peers and nonfamilial adults. Young children with diminished oral language skills present a host of psychosocial adjustment problems (e.g., Salmon et al., 2016). Less is known, however, about factors that amplify risks arising from low oral language proficiency. Considerable attention has focused on the home learning environment, in general, and on an absence of supportive language input, in particular (e.g., Morgan et al., 2015). The present study examines one hitherto unexplored component of the home learning environment: Unsupervised screen time, which typically involves content that does little to promote linguistic, cognitive, or behavioral competence (Korres et al., 2024) and often competes with, distracts from, and reduces higher quality input from adults (Veraska et al., 2021). We test the hypothesis that unsupervised screen time exacerbates socioemotional problems arising from early oral language difficulties in a large community sample of preschool children drawn from childcare centers across Denmark.

We focus here on oral language skills as (opposed to other aspects of language development such as reading comprehension) because of the social language nature of receptive and expressive language development. There are several reasons why diminished oral language skills during early childhood should lead to later socioemotional difficulties. Language abilities are tied to self-regulation and impulse control (Petersen et al., 2014), the lack of which forecast conduct problems (Hill et al., 2006). Poor oral language skills make it difficult to communicate needs; the resulting frustration may lead to sadness or acting out (van der Wilt et al., 2018). Oral language is also key to the development of perspective taking and theory of mind (Siu & Cheung, 2022), which, when delayed, can contribute to adjustment difficulties (Devine et al., 2016) Further, social comparison may diminish self-worth, as children with language difficulties perceive themselves as falling behind classmates (Jerome et al., 2002). Downstream impacts are noteworthy. Conduct problems and emotional problems during the early school years foreshadow a host of later problems, from academic failure and unemployment (Fergusson & Horwood, 1998) to depressive symptoms and substance abuse (Psychogiou et al., 2024).

An emerging consensus holds that excessive electronic media exposure is deleterious to early child development. The World Health Organization recommends less than one hour of screen time per day for children between the ages of 2–5 (WHO, 2019). Yet a recent meta-analysis of global screen time prevalence indicated that 2/3 of households exceed this guideline (McArthur et al., 2022). U.S. parents indicate that half of young children engage with screens for more than 2 h per weekday, with many more exceeding that total on the weekend (Chen & Adler, 2019). Danish parents (the focus of the present study) report that half of 4-year-olds exceed the recommended one-hour daily limit on weekdays, with the vast majority surpassing that number on weekends (Syddansk Universitet, 2024). Such numbers are a source of concern. A wealth of longitudinal evidence ties elevated screen time during early childhood to later emotional and behavioral problems (e.g., Madigan et al., 2019). High exposure to electronic media during early childhood leads to alterations in prefrontal cortex gray matter volume and surface area, with implications for externalizing problems (Paulus et al., 2019). Of course, electronic media content varies in terms of quality, which presumably attenuates or amplifies problematic outcomes. Educational content is assumed to be benign or beneficial (or less deleterious) for very young children, whereas entertainment content has few salutary outcomes (Anthony et al., 2021). We focus here on solitary screen time (defined as content viewed alone) because it is typically associated with more injurious effects than supervised and accompanied media consumption (Korres et al., 2024) and because the bulk of unsupervised screen time is devoted to entertainment as opposed to educational content (Council on Communication and Media, 2013).

Some home learning environments worsen the challenges confronting children with early language difficulties. Unstructured, chaotic homes amplify risks associated with poor language development (Vernon-Feagans et al., 2012). Less obvious but equally challenging are home environments that lack stimulating content or opportunities for supportive interactions. Language-rich home environments are assumed to scaffold the acquisition of learning and social skills, in part by bolstering neurodevelopment; language-limiting home environments are assumed to exacerbate challenges arising from poor language skills because they limit neural plasticity, hindering the acquisition of coping strategies which, in turn, contributes to deficits in emotional regulation and executive functioning (see Zauche et al., 2016). Language-limiting environments come in many forms. Longitudinal associations between language difficulties and child behavior problems, for instance, are amplified in households that infrequently engage in shared book reading (Laursen et al., 2022). Parenting practices, particularly low warmth and high hostility, also increase the risk of child behavior problems tied to early language difficulties (Yew & O’Kearney, 2016).

We suspect that solitary screen time is another—hitherto unexplored—home environment risk factor, amplifying deleterious associations from low language skills to later adjustment difficulties. Young children who are overly-attached to screens miss opportunities to hone social skills or develop proficiencies in activities that promote positive social interactions (Ma et al., 2022). Solitary screen time can result in exposure to inappropriate content, which has been linked to later adjustment problems, in part because improper and inappropriate behaviors are modeled (Fitzpatrick et al., 2012). Finally, screen time has been linked to attention span decrements in young children (Santos et al., 2022), which should amplify the emotional, behavioral, and interpersonal problems of those with language difficulties.

The present study starts from the premise that solitary screen time—like other known home environment risks—both interferes with the adjustment of young children and intensifies socioemotional problems arising from poor oral language skills. We hypothesized that solitary screen time would serve as a main effect predictor of conduct and emotional problems, and a moderator of outcomes arising from oral language difficulties, such that high levels of solitary screen time would amplify the longitudinal association from communication skills and productive vocabulary to increases in conduct and emotional problems. To test these hypotheses, we followed a large community sample of 4- and 5-year-old preschool children for six months. At the outset, parents provided estimates of child solitary screen time and children completed standardized assessments of productive vocabulary and communication skills. Teachers rated child conduct and emotional problems at the beginning and end of the school year. Our goal was to (a) replicate previous findings that tie language difficulties and screen time exposure to later conduct and emotional problems and (b) extend these findings in a new direction by demonstrating how solitary consumption of electronic media exacerbates socioemotional problems among young children with low language skills. The study was conducted in Denmark, which provides a strong test of the hypotheses because all families receive state-provided resources designed to promote child development, which should attenuate risks attached to language-limiting environments.

## Method

### Participants

Participants included 546 (264 girls, 282 boys) 4- and-5-year-olds (*M*_age_=58.8 months, *SD* = 6.8) enrolled in 50 classes in 24 childcare centers from 13 municipalities across Denmark. Parents averaged 14.10 years of formal education (compared to 14.50 years, the national average for adults with preschool-aged children).

### Procedure

Participants were the control groups from two national childcare interventions (Bleses et al., 2018a, b. All 98 Danish municipalities were invited to participate; 13 of 20 that expressed interest were selected to ensure geographic and socioeconomic diversity. Of these municipalities, the 300 childcare centers were stratified by household structure, parent education and income, and use of social services. Each center was then randomly assigned to one of four conditions, including a business-as-usual or control group that did not participate in any of the intervention activities. There were no statistically significant differences between groups on any demographic or any study variables. Teachers (*n* = 50) completed an online questionnaire describing child behavior problems in November 2012 (Fall/Time 1) and June 2013 (Spring/Time 2). In the Time 1, teachers administered standardized language assessments to children in a quiet setting in the classroom. Classroom teachers (at least two per classroom) completed and administered instruments; the same teacher did not necessarily complete or administer surveys to students, within or between time points. Also in the Time 1, parents completed a home environment inventory online. The two time points coincide with the pre- and post-test assessments for the groups that were included in the intervention.

### Instruments

All items for the socioemotional and screen time instruments are included in the appendix. Oral language survey items from national assessments are not available for distribution but may be accessed upon request from the Ministry of Education. (Appendix Table 1).

#### Child Socioemotional Adjustment

 Teachers completed the Danish version (Niclasen et al., 2012) of the Strengths and Difficulties Questionnaire (Goodman, 1997). Items were rated on a scale ranging from 0 (not true) to 2 (certainly true). *Conduct problems* include 5 items (alpha = 0.67) that measure disruptiveness and nonconformity. *Emotional problems* include 5 items (alpha = 0.71) that measure internalizing symptoms. For each variable, item scores were averaged. The recommended 90th percentile score yielded the same clinical cutoffs as the original British sample (Goodman, 1997). Using these cutoffs, 10.6% scored in the clinical range (4 or above) for conduct problems, and 6.4%. scored in the clinical range (5 or above) for emotional problems.

#### Child Oral Language Skills

 *Productive vocabulary* was measured with a 76-item inventory (alpha = 0.97) from a standardized instrument [e.g., What is this? (a picture of an octopus)] that was part of a national assessment administered by educators to all preschool children in Denmark (Højen et al., 2022). Scores represent the sum of correct answers. *Communication skills* measured child language abilities in context with a 10-item (alpha = 0.92) teacher-report inventory (Bleses et al., 2018b) that was also part of the national assessment of preschool children (e.g., How often does the child maintain brief conversations about food when helping prepare lunch in the kitchen?). Items were rated on a scale ranging from 1 (*never*) to 4 (*always*). National policy identifies the 5th percentile (based on a large, representative norming sample) as a cutoff for referral to a speech-language therapist and intervention, with the 6th −15th percentiles as borderline, requiring heightened awareness and initiatives to support the children’s language development within the childcare facilities. Using these cutoffs, 7.69% scored in the clinical range for productive vocabulary and 11.53% scored in the borderline range; 6.52% scored in the clinical range for communication skills and 10.87% scored in the borderline range.

#### Child Solitary Screen Time

Parents (typically the mother) completed a home environment survey designed to capture features that influence early language development (Bleses et al., 2014). *Solitary screen time* was measured with the average of 2 items that assessed the frequency of solo electronic device use during the past week. Items were rated on a scale ranging from 1 (*never*) to 7 (*20–30 h)*, with a midpoint of 4 (3–5 h). Spearman-Brown coefficients, recommended for two-item scales (Eisinga et al., 2012), indicated acceptable reliability (ρ = 0.55).

#### Potential Confounding Variables

 Child oral language skills (Hoff et al., 2012 and screen time use (Tadon et al., 2012) are known to correlate with parent SES. Data protection laws restrict access to individual-level SES data, so the use of books in the home was used instead. A measure of the home learning environment captures much of the variance that would otherwise be assigned to parent education and income (Heppt et al., 2022). *Number of books in the household* (i.e., how many total books do you have in your house?) was rated on a scale ranging from 1 (0–10) to 6 (more than 200).

### Plan of Analysis

Preliminary analyses were conducted using SPSS v.30. correlations between Time 1 and Time 2 variables. These included within time intercorrelations, and 2 (child sex) by 2 (child age) ANOVAs, separately for the predictor variables in the regression.

Two sets of multiple linear regression analyses tested the hypothesis that solitary screen time exacerbates longitudinal associations from low oral language skills to increases in socioemotional problems. Analyses were conducted in Mplus 8.4 (Muthén & Muthén, 2017) with Bayes estimation. Separate analyses were conducted with Time 2 conduct problems and Time 2 emotional problems as dependent variables. The first step in each regression included six Time 1 direct effects predictors: (a) child sex; (b) child age; (c) communication skills; (d) productive vocabulary; (e) solitary screen time; and (f) conduct problems or emotional problems. In the second step, two Time 1 interaction terms were added: (a) solitary screen time $\:\times\:$ communication skills and (b) solitary screen time $\:\times\:$ productive vocabulary. Statistical significance in a Bayesian framework requires *p* <.05 and a 95% credible interval that does not cross 0. Effects are considered marginally significant if (a) *p* <.10 or (b) a *p* <.05 is accompanied by a 95% credible interval that crosses 0 (Gelman et al., 2013).

Follow-up simple slope analyses for statistically significant two-way interactions (Preacher et al., 2006) were plotted at low (1 *SD* below the mean) and high (1 *SD* above the mean) levels of solitary screen time. The procedure estimates slopes at given levels of the predictor, thus utilizing the entire sample without dichotomizing groups.

An average of 1.02% of teacher-report data (i.e., oral language skills) and 31.50% of parent-report data (i.e., socioemotional adjustment and solitary screen time) were missing. There were no statistically significant differences between participants with and without complete data on any study variable. A normed chi square (χ^2^ = 1.98) indicated that data were missing at random and that multiple imputation techniques were appropriate (Bollen, 1989), so missing data were imputed using an EM algorithm with 20 iterations.

Separate Monte Carlo simulations (Muthén & Muthén, 2002) with 10,000 replications were conducted to determine whether there was adequate power (80%) to detect small (β = 0.10), medium (β = 0.30), and large (β = 0.50) effects. There was sufficient power to detect large and medium direct and moderated effects (*M*_power_>0.99, range > 0.99–0.99), but less power to detect small direct effects (*M*_power_=0.65, range = 0.60–0.70) and small moderated effects (*M*_power_=0.69, range = 0.69–0.69).

## Results

### Preliminary Analyses

Table 1 presents descriptive statistics and interclass correlations. Emotional problems and conduct problems were statistically significantly (*p* <.01) positively correlated as were their autocorrelations. Conduct problems and emotional problems were positively correlated with solitary screen time and negatively correlated with communication skills and productive vocabulary. Communication skills and productive vocabulary were positively correlated.

**Table 1:** Correlations, means and standard deviations

Fall (Time 1) Variables	Spring (Time 2) Variables					
	1	2	3	4	5	Mean (SD) [Min, Max]
1. Communication Skills	-					
2. Productive Vocabulary	0.602** [0.545, 0.653]	-				
3. Solitary Screen Time	−0.017 [−0.101, 0.067]	0.039 [−0.045, 0.123]	-			
4. Conduct Problems	− 0.261** [−0.337, −0.181]	− 0.192** [−0.272, −0.110]	0.091* [0.008, 0.174]	0.584** [0.526, 0.637]	0.125** [0.042, 0.207]	0.80 (0.90) [0.00, 2.00]
5. Emotional Problems	− 0.172** [−0.252, −0.090]	− 0.099* [−0.181, −0.015]	0.114** [0.030, 0.196]	0.168** [0.085, 0.248]	0.432** [0.362, 0.498]	0.84 (0.90) [0.00, 2.00]
Mean (*SD*) [Min, Max]	28.94 (6.80) [10.00, 40.00]	50.56 (14.90) [0.00, 74.00]	1.80 (1.30) [0.00, 11.25]	0.83 (0.90) [0.00, 2.00]	0.98 (0.90) [0.00, 2.00]	

*N* = 546. Fall (Time 1) results are below the diagonal and Spring (Time 2) results are above the diagonal, with autocorrelations on the diagonal. Communication skills was rated on a scale ranging from 1 (*never*) to 4 (*always*). Productive vocabulary describes the number of correct items (out of 76). Solitary screen time was rated on a scale ranging from 1 (*never*) to 7 (*20–30 h*). Conduct problems and emotional problems were rated on scales ranging from 0 (*not true*) to 2 (*certainly true*). **p <.01. *p <.05

Separate 2 (child sex) by 2 (child age) ANOVAs were conducted with Time 1 communication skills, Time 1 productive vocabulary, and Time 1 solitary screen time as the dependent variables. There were statistically significant main effects of child age for communication skills and productive vocabulary. In each case, older children scored higher than younger children (*d* = 0.33–0.81). There were also significant main effects of sex on productive vocabulary and solitary screen time. Productive vocabulary was higher for girls than boys (*d =* 0.16), whereas solitary screen time was greater for boys than girls (*d =* 0.23).

### Longitudinal Associations from Oral Language Skills To Socioemotional Adjustment, Moderated by Solitary Screen time

#### Conduct Problems

 The left column of Table 2 presents results from the regression analysis with conduct problems as the dependent variable. There was a statistically significant (*p* <.05) main effect for child sex. Boys had more conduct problems than girls. There were no other main effects.

**Table 2:** Child oral language and solitary screen time as predictors of change in socioemotional adjustment: results from regression analyses

Fall (Time 1) Predictor Variable	Spring (Time 2) Outcome Variable			
	Conduct Problems		Emotional Problems	
	*B*	*p*	*B*	*p*
Gender	0.133	< 0.001	−0.030	0.212
	[0.059, 0.200]		[−0.109, 0.045]	
Age (in years)	−0.031	0.294	0.011	0.316
	[−0.095, 0.052]		[−0.062, 0.103]	
Communication Skills	−0.082	0.032	−0.176	< 0.001
	[−0.146, 0.006]		[−0.246, −0.074]	
Productive Vocabulary	0.047	0.114	0.015	0.314
	[−0.033, 0.143]		[−0.069, 0.123]	
Conduct Problems	0.528	< 0.001		
	[0.465, 0.600]			
Emotional Problems			0.391	< 0.001
			[0.320, 0.467]	
Solitary Screen Time	0.031	0.156	0.081	< 0.001
	[−0.013, 0.095]		[0.026, 0.148]	
Communication Skills x Solitary Screen Time	0.358	0.014	0.004	0.100
	[0.054, 0.708]		[−0.003, 0.014]	
Productive Vocabulary x Solitary Screen Time	−0.509	< 0.001	−0.002	0.248
	[−0.835, −0.223]		[−0.006, 0.002]	

* N =* 546. Standardized beta weights reported with 95% credible intervals in brackets. Child sex: girls=−0.5, boys = 0.5

**Fig. 1:**
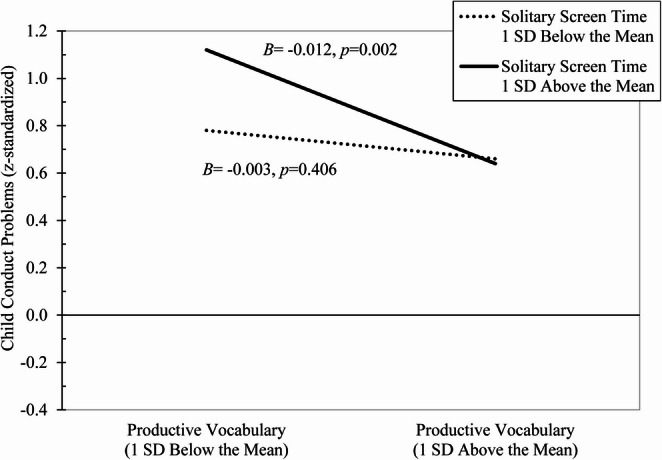
Low initial productive vocabulary predicts heightened subsequent conduct problems at high levels of solitary screen time but not low

There was a statistically significant two-way interaction for solitary screen time x productive vocabulary. Figure 1 indicates that the association between initial productive vocabulary and subsequent conduct problems was moderated by solitary screen time, such that low productive vocabulary predicted increases in conduct problems, but only at high levels of solitary screen time.

**Fig. 2:**
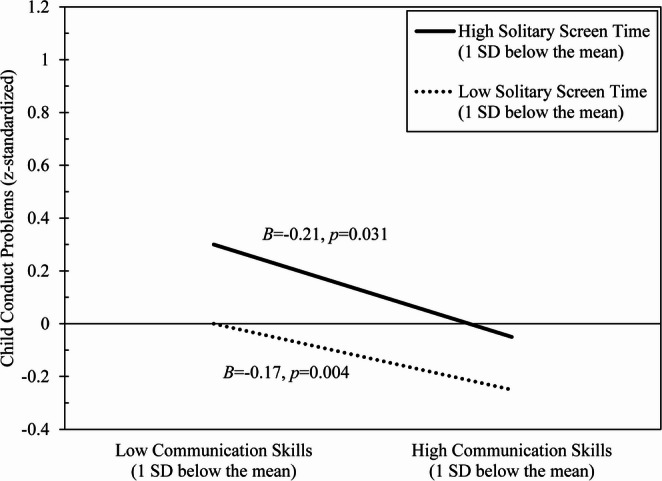
Low initial communication skills predict heightened subsequent conduct problems, with associations stronger at high levels of solitary screen time than at low

There was a borderline statistically significant (*p* <.05, with a credibility interval that crossed zero) main effect for Time 1 communication skills, which was qualified by a statistically significant two-way interaction for solitary screen time x communication skills. Figure 2 indicates that the association between initial communication skills and subsequent conduct problems was moderated by solitary screen time, such that low communication skills predicted greater increases in conduct problems when accompanied by high solitary screen time than when accompanied by low solitary screen time.

#### Emotional Problems

 The right column of Table 2 presents results from the regression analysis. There were statistically significant main effects for Time 1 communication skills and Time 1 solitary screen time. Lower initial communication skills predicted greater increases in emotional problems. Higher initial solitary screen time predicted greater increases in emotional problems.

Main effects for solitary screen time and communication skills were moderated by a borderline statistically significant (*p* <.05, with a credibility interval that crossed zero) interaction for solitary screen time x communication skills. This trend is illustrated in Supplemental Fig. 1, which suggests that low communication skills were associated with greater increases in emotional problems when accompanied by high solitary screen time than when accompanied by low solitary screen time.

## Supplementary Analyses

Parent report of number of books in the home was added to the regression model to control for the contribution of the home learning environment to child oral language skills and solitary screen time. Supplemental Table 1 presents the results. The same pattern of statistically significant results emerged, with three exceptions. First, child age (originally nonsignificant) emerged as a statistically significant main effect predictor of Time 2 conduct problems. Second, Time 1 communication skills dropped to borderline statistical significance (*p* =.066) as a predictor of Time 2 conduct problems. Third, Time 1 productive vocabulary (originally nonsignificant) emerged as a borderline statistically significant predictor of Time 2 conduct problems. Time 1 books in the home did not predict Time 2 conduct problems or emotional problems.

## Discussion

Our findings, drawn from a large representative sample of Danish preschool children, extend previous work on the adjustment correlates of early language difficulties. Unique to our study are results indicating that, over the course of a school year, solitary screen time worsened longitudinal associations from oral language difficulties to subsequent increases in conduct problems. Although solitary screen time also forecast later increases in emotional problems, it did not reliably moderate internalizing problems arising from oral language difficulties, suggesting that amplification mechanisms may be stronger for or limited to conduct problems.

Early language problems are an important harbinger of poor psychosocial adaptation. Our findings align with previous studies that describe the adjustment correlates of poor communication skills. Children who start school with communication difficulties struggle to catch up with their peers. They select classmates as friends who are equally low performing, which inhibits academic performance (Kiuru et al., 2017). Their language problems are quickly compounded by accumulating interpersonal and behavioral challenges (Matte-Landry et al., 2020). A downward spiral unfolds across the primary school years, as low language skills beget academic underperformance (Bleses et al., 2016) and peer rejection (Laursen et al., 2021), which further feed the internalizing and externalizing problems that initially followed from oral language difficulties (Yew & O’Kearney, 2012).

Findings from the present study matter because they indicate that an all-too-common environmental risk–elevated solitary screen time–exacerbates the conduct and behavioral challenges confronting those who face an already difficult developmental path. Screens dominate free time (Konca, 2021), crowding out play and physical activity and, when consumed alone, reduce the kind of social engagement with parents, siblings, and peers that can bolster emotional regulation abilities and emerging social skills (Ma et al., 2022). The entertaining content that most children prefer is brief, fast-paced, and arousing (Bhat, 2017), with the prospect of age-inappropriate content. More fundamentally, some findings suggest that excessive screen exposure effects the prefrontal cortex in ways that give rise to heightened externalizing problems (Paulus et al., 2019). In sum, language difficulties—in this case, poor communication skills and low productive vocabulary—are a well-established risk for conduct problems; unsupervised screen time forecloses opportunities for social engagement that might mitigate this risk.

Like others (Yew & O’Kearney, 2012), we found that poor communication skills predict the development of emotional problems. Social comparison and negative feedback may adversely impact the self-worth of children with language difficulties, eliciting anxiety about and avoidance of social situations. We are also not the first to report that electronic media exposure forecasts emotional problems (Ehrich et al., 2022). Why this is the case for young children is not clear; hypothesized mechanisms include unsettling content, sleep disturbance, and neurobiological disruptions. Although we hypothesized that these two risk factors would converge, findings for the interaction between communication skills and solitary screen time did not rise to conventional levels of statistical significance. Given modest statistical power to detect small moderated effects, we will refrain from interpreting the borderline statistically significant interaction that emerged and simply note that although both variables uniquely predicted the growth of emotional problems, caution is warranted about drawing conclusions as to whether one amplifies problems tied to the other.

The debate over the utility of screen time as a predictor of adjustment outcomes is fraught and complicated by developmental considerations. We cannot resolve it here. Solitary screen time is an imperfect window into the type of content consumed. Previous studies note that unsupervised screen time tends to be dominated by non-educational content (Council on Communication and Media, 2013). Assuming the same applies to the solitary screen time reported herein, it could be argued that our findings underestimate the correlates of unsupervised entertainment content because some parents undoubtedly constrained solitary viewing choices to educational material. We suspect that content restrictions on unsupervised media consumption will vary across households, driven by parent education and household resources. Future studies that aim to assess the developmental consequences of elevated screen time should evaluate the type of media consumed and whether it is consumed alone or in the company of others. Recent findings (Lu & Li, 2025) indicating that the impact of screen time is mitigated by joint engagement and the quality of the home environment underscore the need for caution in overly proscriptive injunctions against media exposure.

We hasten to note that electronic media, consumed in moderation, is not necessarily deleterious and may even be beneficial. It is important to recognize, however, that consequences differ as a function of age, as do recommendations concerning exposure. Health organizations consistently define excessive screen time as more than one hour per day for 2–5-year-olds, regardless of content (e.g., WHO, 2019). There are several reasons offered for limiting exposure to screens during early childhood. Emerging evidence points to neurological costs for the very young (e.g., Adams et al., 2023), although much remains unknown on this point. Clearer are the opportunity costs: Time spent alone with a screen is time that is not spent with friends and family members, time that is not spent sleeping, playing or engaged in physically robust activities. Our study adds to this list of concerns. Young children learn language more effectively from in-person interactions than from video screens (Roseberry et al., 2009). Solitary screen time among preschool-aged children may be uniquely unhelpful for those with oral communication challenges, in part because it may have few benefits for those whose language skills are lagging, in part because it is devoid of the dialogic components that foster communication skills and provide opportunities for explanation and elaboration (Massaroni et al., 2023), and in part because it interferes with the development of executive function and emotional regulation skills necessary for successful social engagement (McHarg et al., 2020).

Our study is not without limitations. As noted above, the solitary screen time survey did not address the type of content consumed. Firm conclusions about the risks of specific content exposure should be avoided. Relatedly, solitary screen time was assessed through parent reports. It is not clear how accurate parents are in their reporting efforts, but we suspect they are more likely to underestimate solitary screen time than to overestimate it. Productive vocabulary was a standardized instrument, suggesting that teacher-report bias was not a factor. The same cannot be said for communication skills and indices of socioemotional adjustment, raising the possibility of unmeasured classroom level variability. It is not clear, however, that teacher-report biases would systematically impact reports, particularly given that more than one teacher per class completed surveys and administered assessments. The impact on our results depends on whether there are systematic differences in how much and for whom the reporting errors involved. Multilevel modeling is needed to address nestedness, but our classrooms contained too few students for these analyses (Peugh, 2010). Socioeconomic status is known to correlate with both screen time (Tadon et al., 2012) and language development (Hoff et al., 2012); controlling for SES helps to isolate effects to the variables on interest. Privacy laws prevented us from linking SES data to these results, so we used books in the home instead, a measure of the home learning environment that captures much of the variance that would otherwise be assigned to parent education and income (Heppt et al., 2022). Finally, the sample was representative of children in Denmark, where state support ensures that families enjoy a relatively high minimum standard of living. The findings may well be stronger in less advantaged contexts where resources for young children with language and socioemotional difficulties are not as easy to attain.

Young children with poor language skills are prone to socioemotional problems. Solitary screen time appears to make matters worse, amplifying conduct problems tied to oral language difficulties. We argue that electronic media should be regarded as an integral component of the home learning environment; many children spend more time with tablets and phones than with toys, books, and friends. Like other home environment risks, solitary screen time poses a unique peril to young children with heightened vulnerabilities. There is little reason to expect that screens help children overcome the adaptive challenges posed by oral language problems and many reasons to suspect that they make matters worse. 

## Supplementary Information

Below is the link to the electronic supplementary material.

Supplementary Material 1 (DOCX. 17.6 KB)

## Appendix

**Appendix Table 1:** Variable items

Variable	Item 1	Item 2	Item 3	Item 4	Item 5
Conduct Problems	Often fights with/bullies other children	Often lies or cheats	Steals from home, school or elsewhere	Often has temper tantrums	Is generally obedient (or usually does what adult requests)
Emotional Problems	Often unhappy, downhearted or tearful	Complains of headaches, stomach aches or sickness	Many worries, often seems worried	Nervous or clingy in new situations, easily loses confidence	Many fears, easily scared
Solitary Screen Time	How many hours has your child watched TV or movies on a tablet, computer, phone, television, alone?	How many hours has your child played games on a tablet, computer, phone, television or game consol, alone?			
Number of Books in the Home	How many total books do you have in your house?				

Conduct Problems and Emotional Problems were rated on a scale ranging from 0 (not true) to 2 (certainly true). Solitary Screen Time was rated on a scale ranging from 1 (never) to 7 (20–30 h). Number of Books in the Home was rated on a scale ranging from 1 (0–10) to 6 (more than 200)
