# Supplementary material for: Solitary Screen Time Exacerbates Later Socioemotional Problems in Young Children with Oral Language Difficulties
Source: Res Child Adolesc Psychopathol. 2026 Mar 12;54(2):44. doi: 10.1007/s10802-025-01409-8 (PMC12982259; doi:10.1007/s10802-025-01409-8)
Supplement: Supplementary file 1 — Supplementary Material 1 (DOCX. 17.6 KB) [file 10802_2025_1409_MOESM1_ESM.docx]

**Supplemental Table**. *Child Oral Language and Solitary Screen Time as Predictors of Change in Socioemotional Adjustment: Results from Supplemental Regression Analyses Including Number of Books in the Home as a Control Variable.*

|  | Spring (Time 2) Outcome Variables | | | |
| --- | --- | --- | --- | --- |
|  | Conduct Problems | | Emotional Problems | |
| Fall (Time 1) Predictor Variables | B | *p* | B | *p* |
| Gender | 0.127 | <0.001 | -0.036 | 0.196 |
|  | [0.062, 0.192] |  | [-0.113, 0.032] |  |
| Age (in years) | -0.075 | <0.001 | 0.023 | 0.272 |
|  | [-0.141, -0.003] |  | [-0.050, 0.099] |  |
| Communication Skills | -0.074 | 0.066 | -0.159 | <0.001 |
|  | [-0.139, 0.007] |  | [-0.231, -0.072] |  |
| Productive Vocabulary | 0.061 | 0.082 | 0.006 | 0.418 |
|  | [-0.021, 0.192] |  | [-0.086, 0.148] |  |
| Conduct Problems | 0.525 | <0.001 |  |  |
|  | [0.455, 0.566] |  |  |  |
| Emotional Problems |  |  | 0.384 | <0.001 |
|  |  |  | [0.316, 0.441] |  |
| Number of Books in the Home | -0.038 | 0.112 | -0.002 | 0.496 |
|  | [-0.085, 0.033] |  | [-0.055, 0.074] |  |
| Solitary Screen Time | 0.034 | 0.170 | 0.085 | 0.004 |
|  | [-0.033, 0.104] |  | [0.008, 0.158] |  |
| Communication Skills  x Solitary Screen Time | 0.261 | 0.040 | 0.118 | 0.234 |
|  | [-0.022, 0.557] |  | [-0.203, 0.431] |  |
| Productive Vocabulary  x Solitary Screen Time | -0.464 | <0.001 | -0.097 | 0.162 |
|  | [-0.710, -0.215] |  | [-0.373, 0.191] |  |

*Note.* *N=*546. Standardized beta weights reported with 95% credible intervals in brackets. Child sex: girls=-0.5, boys=0.5.
